# Influence and risk factors of postoperative infection after surgery for ischemic cardiomyopathy

**DOI:** 10.3389/fcvm.2023.1231556

**Published:** 2023-08-24

**Authors:** Bing Wen, Yang Lu, Xiaofan Huang, Xinling Du, Fuqiang Sun, Fei Xie, Chao Liu, Dashuai Wang

**Affiliations:** ^1^ Department of Cardiovascular Surgery, The First Affiliated Hospital of Zhengzhou University, Zhengzhou, China; ^2^ Department of Cardiology, The First Affiliated Hospital of Zhengzhou University, Zhengzhou, China; ^3^ Department of Cardiovascular Surgery, Union Hospital, Tongji Medical College, Huazhong University of Science and Technology, Wuhan, China

**Keywords:** postoperative infection, heart failure, ischemic cardiomyopathy, coronary artery disease (CAD), coronary artery bypass graft (CABG)

## Abstract

**Background:**

Studies on postoperative infection (POI) after surgery for ischemic cardiomyopathy are still lacking. This study aimed to investigate the risk factors of POI and its influence on clinical outcomes in patients undergoing ischemic cardiomyopathy surgery.

**Methods:**

The Surgical Treatment for Ischemic Heart Failure (STICH) trial randomized patients with ischemic cardiomyopathy [coronary artery disease (CAD) with left ventricular ejection fraction ≤35%] to surgical and medical therapy. In this study, a *post hoc* analysis of the STICH trial was performed to assess the risk factors and clinical outcomes of POI in those undergoing coronary artery bypass graft (CABG). Patients were divided according to whether POI developed during hospitalization or within 30 days from operation.

**Results:**

Of the 2,136 patients randomized, 1,460 patients undergoing CABG per-protocol was included, with a POI rate of 10.2% (149/1,460). By multivariable analysis, POI was significantly related to patients' age, body mass index, depression, chronic renal insufficiency, Duke CAD Index, and mitral valve procedure. Compared to patients without POI, patients with POI had significantly longer durations of intubation, CCU/ICU and hospital stay, and higher rates of re-operation, in-hospital death and failed discharge within 30 days postoperatively. In addition, these patients had significantly higher risks of all-cause death, cardiovascular death, heart failure death, and all-cause hospitalization during long-term follow-up. However, the influence of POI on all-cause death was mainly found during the first year after operation, and the influence was not significant for patients surviving for more than 1 year.

**Conclusions:**

POI was prevalent after surgery for ischemic cardiomyopathy and was closely related to short-term and long-term clinical outcomes, and the effect of POI mainly occurred within the first postoperative year. This study first reported and clarified the relationship between POI and long-term prognosis and the predictors for POI after surgery for ischemic cardiomyopathy worldwide, which may have certain guiding significance for clinical practice.

**Clinical Trial Registration:**

https://www.clinicaltrials.gov, identifier (NCT00023595).

## Introduction

As a global pandemic with an increasing prevalence, heart failure (HF) remains a leading cause of mortality and morbidity (1–3). Ischemic cardiomyopathy, defined as severe coronary artery disease (CAD) with left ventricular (LV) dysfunction, is the most common cause of HF with reduced ejection fraction (EF) (3, 4). Despite advances in diagnosis and medical management these years, surgical revascularization for ischemic cardiomyopathy using coronary artery bypass graft (CABG) is still recommended as the preferred treatment strategy (4, 5). Nevertheless, the overall survival after surgery remains unsatisfied and the rates of various postoperative complications remain high (6).

Postoperative infection (POI) is one of the most prevalent complications after cardiac surgery, related to inferior outcomes and increased resource use (7–9). The incidence of POI varies widely in previous reports due to different definitions, specific types of infections, and surgical populations in different studies (9–12). Multiple types of infections have been reported in the literature, such as mediastinitis, surgical wound infection, pneumonia and sepsis (9–14). Regardless of the infectious type, the development of POI will increase mortality, prolong hospital stay, and increase total treatment costs to varying degrees. Compared to the majority of the common cardiac surgery, patients undergoing ischemic cardiomyopathy surgery had significantly higher perioperative risk due to the inherently more severe and complex nature of the condition. It is precisely due to the complexity and high risk of the ischemic cardiomyopathy surgery that has led to the fact that there were still few studies specifically conducted in this patient population. Notably, studies focused on POI conducted in patients undergoing surgery for ischemic cardiomyopathy are still lacking so far.

The Surgical Treatment for Ischemic Heart Failure (STICH) study was a large-scale, multicenter, randomized controlled trial that enrolled 2,136 patients with coronary artery disease (CAD) amenable to CABG and left ventricular ejection fraction (LVEF) ≤35% (15). Using data from the STICH study and the STICH extension study (STICHES), we aimed to (1) identify the risk factors of POI after CABG for ischemic cardiomyopathy, (2) assess the influence of POI on short-term (in-hospital or within 30 postoperative days) clinical outcomes, and (3) assess the influence of POI on long-term follow-up outcomes.

## Methods

This study is a *post hoc* analysis of the STICH/STICHES study (15, 16) using data from the National Heart, Lung, and Blood Institute's Biologic Specimen and Data Repository Information Coordinating Centre (BioLINCC). This study complies with the Declaration of Helsinki and an exemption from the institutional review board oversight was granted due to the deidentified nature of the data. Written informed consent was obtained from each included participant and the study protocols were approved by the institutional review board at each site.

### Study population

Study details with regard to the rationale, design, enrolment, and main findings of the STICH/STICHES trial have been published previously (15–17). Briefly, a total of 2,136 patients with ischemic cardiomyopathy from 127 clinical sites in 4 continents and 26 countries between 2002 and 2007 were enrolled to test two hypotheses. In hypothesis 1, 1,212 patients were enrolled and randomized to either medical therapy + CABG (*n* = 610) or medical therapy alone (*n* = 602). In hypothesis 2, 1,000 patients were included and randomized to undergo CABG + surgical ventricular reconstruction (SVR) (*n* = 501) or CABG alone (*n* = 499). Among the 2,136 patients enrolled in the STICH/STICHES trial, the following criteria were used to exclude participants from this study: (1) patients were randomized to the medical therapy group (*n* = 602); (2) patients were randomized to but did not receive surgical treatment actually in both hypothesis 1 and 2 (*n* = 74). Patients randomized to medical therapy but received CABG during the follow-up period (*n* = 65) were also excluded for this study due to the fact that detailed perioperative and postoperative data were not available in these patients. The remaining 1,460 patients met the inclusion criteria and were included for further analysis in this study.

### Endpoints, variables, and outcome events

In this study, the primary endpoint was POI within 30 days after surgery for ischemic cardiomyopathy. POI was defined as the development of any type of major postoperative infections, such as mediastinitis, pneumonia, pyelonephritis, septicemia, and infections at the vein-harvest site, just as defined in previously published literature (6). Mediastinitis was defined as any wound disruption exposing the sternum or requiring a secondary operation or stabilization of the sternum; pneumonia was defined as infection of the lung often accompanied by inflammation; pyelonephritis was defined as inflammation of the kidney involving the renal parenchyma; septicemia was defined as systemic inflammatory response syndrome with a proven or suspected infectious etiology. Patients were divided into two groups according to whether POI developed and pre-, intra- and post-operative variables and outcomes were compared between the two groups.

After selection, preoperative variables analyzed in this study included sex, age, race, body mass index, body surface area, current smoker, depression, hypertension, diabetes, hyperlipidemia, myocardial infarction, stroke, peripheral vascular disease, atrial fibrillation/flutter, chronic renal insufficiency, previous percutaneous coronary intervention (PCI), previous CABG, Canadian Cardiovascular Society (CCS) angina class, New York Heart Association (NYHA) class, LVEF, number of diseased vessels, left main stenosis, proximal left anterior descending artery (LAD) stenosis, Duke CAD Index, hemoglobin, serum creatinine and mitral regurgitation. Intraoperative variables included off/on-pump bypass, concomitant SVR, mitral valve procedure, cardiopulmonary bypass (CPB) time, and aortic cross clamp time. Postoperative variables before discharge or within 30 days after surgery included the durations of intubation, cardiac or intensive care unit (CCU/ICU) and hospital stay, and the incidence rates of re-operation, in-hospital death and failed discharge within 30 days. All postoperative events and complications has been prespecified and defined in previous reports (6).

All the enrolled patients was followed up at the time of hospital discharge, 30 days after surgery, at 4-month intervals for the first year, and at 6-month intervals over the remainder of the follow-up period thereafter. In this study, the follow-up outcomes included all-cause death, cardiovascular death, heart failure (HF) death, all-cause hospitalization, cardiovascular hospitalization, HF hospitalization, and the composite of all-cause death and all-cause, cardiovascular and HF hospitalization.

### Statistical analysis

Continuous variables were biased and were expressed as median (25th, 75th percentile), and were compared by Mann–Whitney *U*-test. Categorical variables were expressed as count (percentage) and compared by chi-square test or Fisher's exact test. Independent risk factors for POI were identified by multivariable logistic regression analysis with a forward stepwise procedure. The Cox proportional hazards regression model was used to assess the influence of POI on long-term outcomes in both univariable and multivariable analyses. The multivariable model was adjusted for sex, age, race, body mass index, current smoking, depression, hypertension, diabetes, hyperlipidemia, myocardial infarction, stroke, peripheral vascular disease, atrial fibrillation/flutter, chronic renal insufficiency, previous PCI, previous CABG, CCS angina class, NYHA class, left ventricular ejection fraction, Duke CAD Index, hemoglobin, mitral regurgitation, surgical treatment and CPB time. The results were presented as odds ratio (OR) or hazard ratio (HR) with 95% confidence interval (CI). Cumulative event rates were calculated by the Kaplan-Meier (KM) method and the log-rank test was used for comparison between groups.

Two-tailed *P*-values of less than 0.05 were considered statistically significant. All analyses were performed under the per-protocol principle using R software (version 4.0.5) and SPSS (IBM SPSS Statistics 26.0, SPSS Inc., Chicago, IL).

## Results

### Characteristics

After screening, a total of 1,460 patients undergoing CABG per-protocol were included in this study, with a POI rate of 10.2% (149/1,460) within 30 days postoperatively. The median age of these patients was 61.2 (54.0, 68.3) years, 13.7% were female. Compared to patients with POI, patients without POI had younger age, lower depression rate, better renal function, better cardiac function, fewer diseased vessels, higher Duke CAD Index, less on-pump bypass and mitral valve procedure, as well as shorter CPB and aortic cross clamp time. The details of comparison of baseline characteristics and intraoperative variables in patients with and without POI are summarized in Table 1.

**Table 1:** Preoperative baseline characteristics and operative variables in patients with or without POI.

Variable	Without POI, *n* = 1,311 (%)	With POI, *n* = 149 (%)	*P*-value
Female	178 (13.6)	22 (14.8)	0.689
Age (years)	61 (54, 68)	64 (55, 71)	0.007
Ethnic minority	282 (21.5)	30 (20.1)	0.698
Body mass index (kg/m2)	26.9 (24.3, 30.1)	27.3 (24.2, 30.6)	0.080
Body surface area (m2)	1.9 (1.8, 2.1)	1.9 (1.8, 2.1)	0.578
Current smoker	271 (20.7)	33 (22.1)	0.674
Depression	81 (6.2)	16 (10.7)	0.034
Hypertension	775 (59.1)	94 (63.1)	0.349
Diabetes	473 (36.1)	61 (40.9)	0.243
Hyperlipidemia	887 (67.7)	98 (65.8)	0.641
Myocardial infarction	1,088 (83.0)	124 (83.2)	0.943
Stroke	86 (6.6)	11 (7.4)	0.702
Peripheral vascular disease	187 (14.3)	28 (18.8)	0.139
Atrial fibrillation/flutter	146 (11.1)	24 (16.1)	0.073
Chronic renal insufficiency	94 (7.2)	24 (16.1)	<0.001
Previous PCI	214 (16.3)	28 (18.8)	0.443
Previous CABG	32 (2.4)	6 (4.0)	0.249
CCS angina class			0.227
0	370 (28.2)	51 (34.2)	
I	129 (9.8)	15 (10.1)	
II	359 (27.4)	28 (18.8)	
III	375 (28.6)	45 (30.2)	
IV	78 (5.9)	10 (6.7)	
NYHA class			0.002
I	127 (9.7)	9 (6.0)	
II	620 (47.3)	57 (38.3)	
III	511 (39.0)	69 (46.3)	
IV	53 (4.0)	14 (9.4)	
Left ventricular ejection fraction (%)	27.5 (21.8, 33.1)	26.3 (21.7, 30.6)	0.056
Number of diseased vessels (stenosis ≥75%)			0.001
0	28 (2.1)	1 (0.7)	
1	255 (19.5)	23 (15.4)	
2	541 (41.3)	45 (30.2)	
3	487 (37.1)	80 (53.7)	
Left main stenosis ≥50%	179 (13.7)	26 (17.4)	0.206
Proximal LAD stenosis ≥75%	947 (72.2)	111 (74.5)	0.558
Duke CAD Index	65 (39, 77)	71 (52, 91)	<0.001
Hemoglobin (g/dl)	13.7 (12.7, 14.8)	13.8 (12.5, 15.0)	0.958
Serum creatinine (mg/dl)	1.1 (0.9, 1.2)	1.2 (1.0, 1.4)	<0.001
Mitral regurgitation			0.017
None or trace	491 (37.5)	44 (29.5)	
Mild	608 (46.4)	69 (46.3)	
Moderate	175 (13.3)	26 (17.4)	
Severe	37 (2.8)	10 (6.7)	
Off-pump bypass	150 (11.4)	7 (4.7)	0.012
Concomitant SVR	446 (34.0)	49 (32.9)	0.782
Mitral valve procedure	183 (14.0)	43 (28.9)	<0.001
CPB time (minutes)	97 (65, 130)	115 (80, 168)	<0.001
Aortic cross clamp time (minutes)	60 (38, 85)	73 (50, 107)	<0.001

CABG, coronary artery bypass graft; CAD, coronary artery disease; CCS, Canadian Cardiovascular Society; CPB, cardiopulmonary bypass; LAD, left anterior descending artery; NYHA, New York Heart Association; PCI, percutaneous coronary intervention; POI, postoperative infection; SVR, surgical ventricular reconstruction.

### Independent risk factors for POI

Variables with a *P*-value of less than 0.1 in the univariable analysis or considered clinically significant were further analyzed by a multivariable logistic regression procedure to identify independent risk factors for POI. Six factors were identified to be significantly associated with the development of POI by multivariable analysis, including age, body mass index, depression, chronic renal insufficiency, Duke CAD Index, and mitral valve procedure (Table 2).

**Table 2:** Multivariate analysis of significant risk factors for POI.

Characteristic	Coefficient	Standard error	OR (95% CI)	*P*–value
Age (years)	0.024	0.010	1.024 (1.005–1.044)	0.014
Body mass index (kg/m2)	0.042	0.019	1.043 (1.006–1.083)	0.024
Depression	0.621	0.299	1.860 (1.036–3.342)	0.038
Chronic renal insufficiency	0.703	0.257	2.019 (1.220–3.340)	0.006
Duke CAD Index	0.012	0.004	1.012 (1.004–1.021)	0.003
Mitral valve procedure	0.924	0.203	2.519 (1.692–3.750)	<0.001
Intercept	−5.960	0.908	0.003	<0.001

CAD, coronary artery disease; CI, confidence interval; OR, odds ratio; POI, postoperative infection.

### In-hospital or 30-day postoperative outcomes

Clinical outcomes in hospital or within the first 30 days postoperatively are compared and summarized in Table 3. The overall 30-day mortality after surgery was 5.1% (74/1,460), with a rate of 9.4% in patients with POI vs. 4.6% in those without POI (*P *= 0.011). A total of 77 patients (5.3%) had a hospital stay of longer than 30 days, while the rate was significantly higher in patients with POI (1.8% vs. 35.6%, *P *< 0.001). Higher rate of re-operation was also observed in patients with POI (5.0% vs. 24.2%, *P *< 0.001). In addition, the durations of total intubation time, CCU/ICU and hospital stay were also significantly prolonged in patients with POI (Table 3).

**Table 3:** Comparison of in-hospital or 30-day postoperative outcomes in patients with or without POI.

Outcome	Without POI, *n* = 1,311 (%)	With POI, *n* = 149 (%)	*P*-value
Total intubation time (hours)	15.5 (11.2, 22.5)	22.0 (13.9, 48.1)	<0.001
Time in CCU/ICU (hours)	51.2 (38.5, 96.0)	137.3 (65.3, 285.8)	<0.001
Hospital stay (days)	8 (7, 12)	21 (12, 41)	<0.001
Re-operation	65 (5.0)	36 (24.2)	<0.001
Hospital stay longer than 30 days	24 (1.8)	53 (35.6)	<0.001
Postoperative death	60 (4.6)	14 (9.4)	0.011
Death or not discharged within 30 days	84 (6.4)	67 (45.0)	<0.001

CCU, cardiac care unit; ICU, intensive care unit; POI, postoperative infection.

### Long-term follow-up outcomes

**Figure 1:**
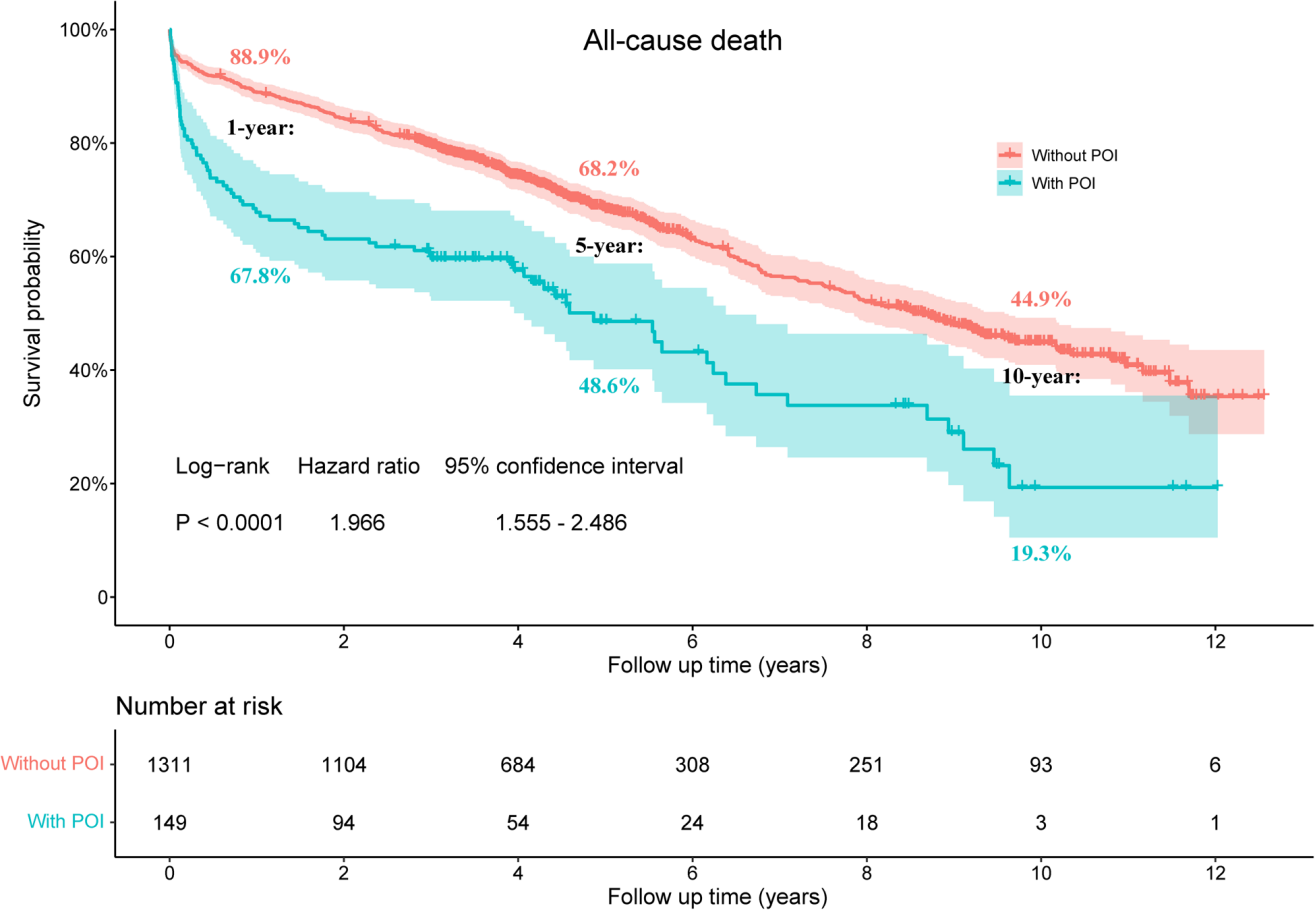
Kaplan-Meier estimated survival probabilities in patients with and without POI. POI, postoperative infection.

**Figure 2:**
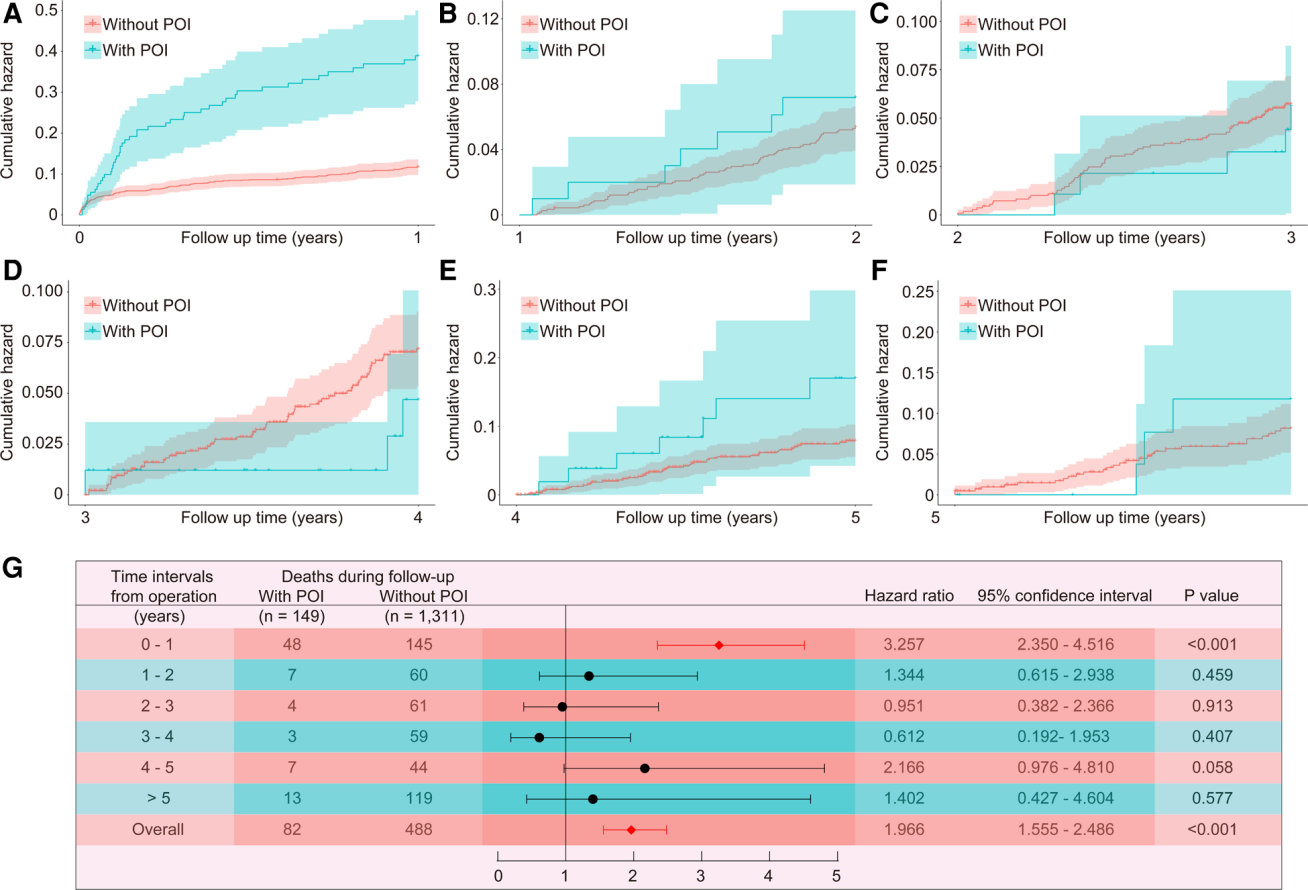
Temporal analysis of the effect of POI on all-cause death. The Kaplan-Meier estimated cumulative incidence of all-cause death during the follow-up year 0–1 (**A**), 1–2 (**B**), 2–3 (**C**), 3–4 (**D**), 4–5 (**E**), >5 (**F**), and time-varying hazard ratios (**G**) in patients with and without POI. POI, postoperative infection.

The median follow-up time of this study population was 4.7 (3.3, 9.4) years, with a maximum follow-up period of 13.3 years. Compared to patients without POI, patients with POI had significantly higher risk of all-cause death throughout the follow-up period (HR: 1.966, 95% CI: 1.555–2.486, *P *< 0.001; Figure 1). This difference remained significant even after adjusting for confounding factors by multivariable Cox proportional hazards regression model (HR: 1.472, 95% CI: 1.148–1.886, *P *= 0.002; Table 4). The KM estimates of survival probabilities of 1-, 5-, and 10-year in patients without POI were respectively 88.9%, 68.2%, and 44.9%; however, the survival probabilities were significantly reduced in patients with POI, with corresponding values of 67.8%, 48.6%, and 19.3%, respectively. To further clarify the impact of POI on postoperative survival, a temporal analysis were performed. The results showed that the effect of POI on the increased risk of postoperative death was mainly reflected in the first postoperative year (HR: 3.257, 95% CI: 2.350–4.516, *P *< 0.001; Figure 2A). However, the effect of POI on the risk of all-cause death was not significant during follow-up one year later (*P *> 0.05; Figures 2B–G).

**Table 4:** Hazard ratios of POI for long-term outcomes by univariable and multivariable Cox regression analysis.

Event	No. of events (KM 10-year rate)		Unadjusted, HR (95% CI)	*P*-value	Adjusted^a^, HR (95% CI)	*P*-value
	Without POI	With POI				
All-cause death	479 (55.1%)	82 (80.7%)	1.966 (1.555–2.486)	<0.001	1.472 (1.148–1.886)	0.002
Cardiovascular death	339 (39.2%)	63 (65.1%)	2.078 (1.588–2.720)	<0.001	1.547 (1.165–2.054)	0.003
Heart failure death	101 (15.8%)	16 (30.3%)	1.888 (1.114–3.200)	0.018	1.225 (0.690–2.175)	0.488
All-cause hospitalization	735 (76.0%)	77 (86.8%)	1.272 (1.006–1.610)	0.045	0.962 (0.753–1.230)	0.757
Cardiovascular hospitalization	570 (63.7%)	59 (75.1%)	1.192 (0.912–1.559)	0.199	0.879 (0.663–1.164)	0.367
Heart failure hospitalization	292 (34.8%)	30 (39.0%)	1.188 (0.816–1.730)	0.369	0.797 (0.536–1.185)	0.261
All-cause death or all-cause hospitalization	933 (85.0%)	125 (95.9%)	1.620 (1.343–1.953)	<0.001	1.232 (1.012–1.500)	0.037
All-cause death or cardiovascular hospitalization	826 (78.6%)	115 (93.4%)	1.568 (1.290–1.907)	<0.001	1.168 (0.950–1.437)	0.140
All-cause death or heart failure hospitalization	620 (64.0%)	96 (86.3%)	1.796 (1.448–2.227)	<0.001	1.332 (1.060–1.675)	0.014

CI, confidence interval; HR, hazard ratio; KM, Kapkan-Meier; POI, postoperative infection.

^a^ Adjusted for sex, age, race, body mass index, current smoking, depression, hypertension, diabetes, hyperlipidemia, myocardial infarction, stroke, peripheral vascular disease, atrial fibrillation/flutter, chronic renal insufficiency, previous PCI, previous CABG, CCS angina class, NYHA class, left ventricular ejection fraction, Duke CAD Index, hemoglobin, mitral regurgitation, surgical treatment and CPB time.

**Figure 3:**
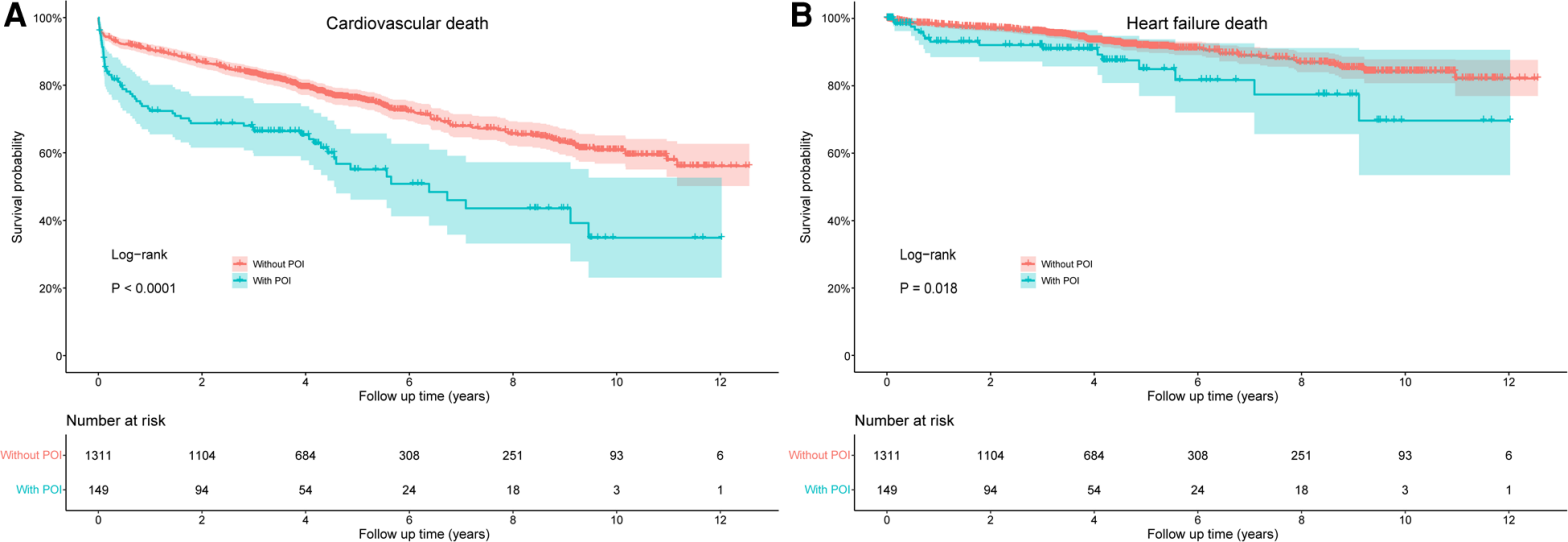
Comparison of cardiovascular death (**A**) and heart failure death (**B**) between patients with and without POI during follow-up. POI, postoperative infection.

**Figure 4:**
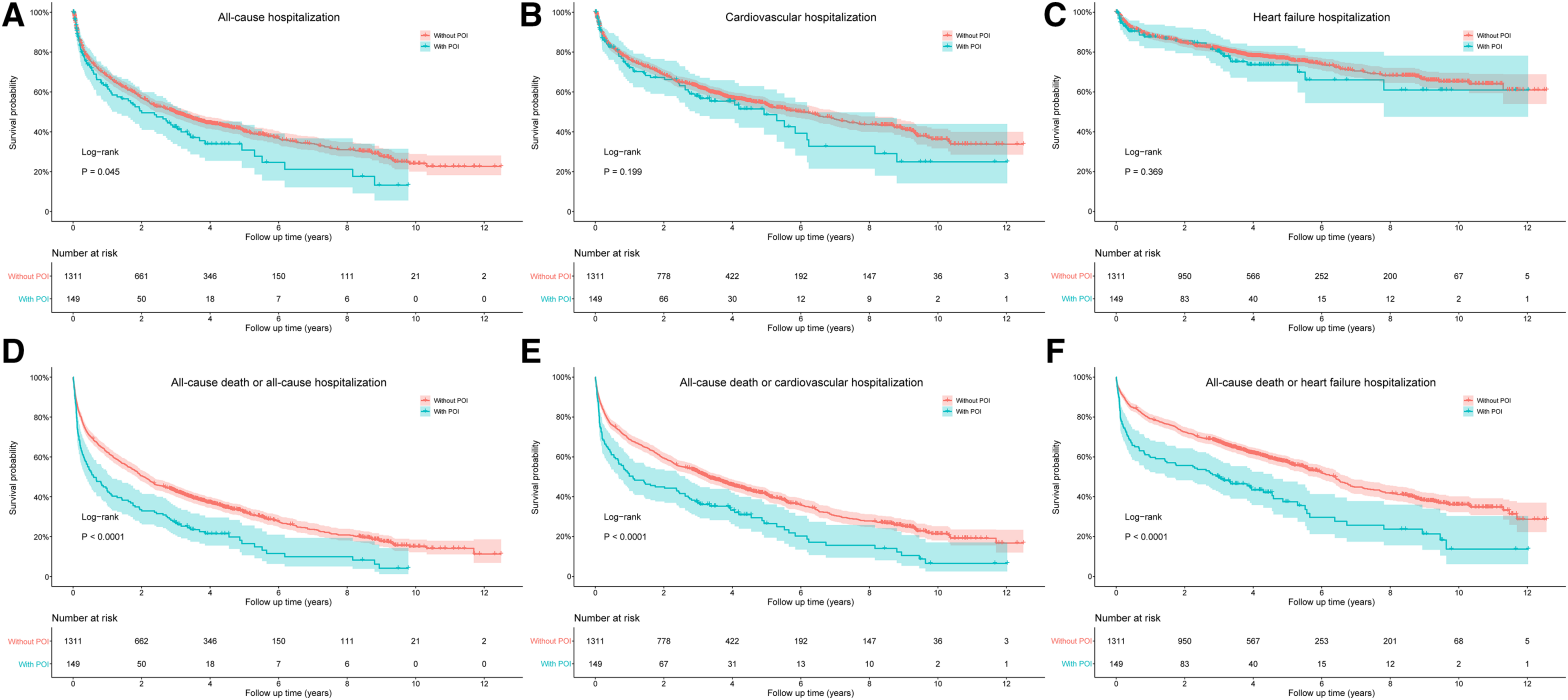
Comparison of all-cause hospitalization (**A**), cardiovascular hospitalization (**B**), heart failure hospitalization (**C**), all-cause death or all-cause hospitalization (**D**), all-cause death or cardiovascular hospitalization (**E**), and all-cause death or heart failure hospitalization (**F**) between patients with and without POI during follow-up. POI, postoperative infection.

The risks of cardiovascular death and HF death were also significantly increased in patients with POI by log-rank test, with HR values of 2.078 (95% CI: 1.588–2.720, *P *< 0.001) and 1.888 (95% CI: 1.114–3.200, *P *= 0.018), respectively (Figure 3). After adjusting for confounding factors, the difference for cardiovascular death between groups remained significant (HR: 1.547, 95% CI: 1.165–2.054, *P *= 0.003), but the difference for HF death disappeared (*P *= 0.488; Table 4). Similarly, patients with POI had higher risk of all-cause hospitalization by log-rank test (HR: 1.272, 95% CI: 1.006–1.610, *P *= 0.045; Figure 4A), but the difference disappeared in multivariable analysis (*P *= 0.757; Table 4). The difference for cardiovascular hospitalization and HF hospitalization was not significant between patients with and without POI by both log-rank test (Figures 4B,C) and multivariable analysis (Table 4).

The impact of POI on the composite endpoint events of all-cause death and all-cause/cardiovascular/HF hospitalization was also evaluated by both log-rank test and multivariable analysis. The results indicated that patients with POI had higher risks of all the three composites by log-rank test (Figures 4D–F), and the difference remained significant for the composites of all-cause death and all-cause/HF hospitalization by multivariable analysis (Table 4).

## Discussion

Infections after cardiac surgery are common and closely associated with higher risks of poor clinical outcomes (7–9), which was confirmed again by our results. In this study, the POI rate after surgery for ischemic cardiomyopathy within the first 30 postoperative days was 10.2%, and the 30-day mortality rate was 5.1%. Six independent risk factors for POI was identified, including age, body mass index, depression, chronic renal insufficiency, Duke CAD Index, and mitral valve procedure. Compared to patients without POI, patients with POI had significantly higher risks of re-operation and postoperative death, and significantly longer durations of total intubation, CCU/ICU, and hospital stay. The risks of all-cause death, cardiovascular death, HF death and all-cause hospitalization were significantly increased in patients with POI, as well as the risks of the composites of all-cause death and all-cause/cardiovascular/HF hospitalization. However, the effect of POI on postoperative death was mainly reflected in the first postoperative year, and was no longer significant during follow-up one year later.

Infections following cardiac surgery is a broad concept that encompasses many different types of infection, such as mediastinitis and pneumonia. Numerous studies focused on both POI and each type of infection have been conducted and reported due to the high prevalence since the introduction of cardiac surgery (9–12, 18). Although considerable progress has been made in drug research and postoperative care in recent years, the incidence of kinds of POI remains high (7, 10, 19–22). A recent multicenter retrospective cohort study conducted by Joseph and colleagues reported that POI developed in 8.0% of the adults undergoing elective CABG, valve surgery or a combination, in which the most common infection was pulmonary, followed by urinary tract, sepsis/bacteremia, wound and gastrointestinal infections (7). The incidence of POI varies dramatically across studies, even for the same type of infection (11). Previous studies indicated that the incidence of POI varies widely in different types of cardiac surgery, with rates of pneumonia 2%–15%, deep sternal wound infection 0.2%–8.0%, and sepsis up to 9.5% (11, 13, 23, 24). The overall incidence of POI in this study was 10.2%, falling within previously reported ranges in the literature.

The six independent risk factors for POI identified in this study was consistent with clinical perception and previous reports. A higher Duke CAD Index represents a higher severity of the lesion and concomitant mitral valve procedure indicates more complex procedures and injuries, which may both increase the risks of various adverse outcomes (25). Advanced age has been widely identified to be associated with the development of various infections after kinds of surgeries, such as pneumonia after acute aortic dissection, heart valve, and redo cardiac surgery (25). This may be due to the fact that with the increase of age, patients may have more comorbidities, declined organ function, weakened defense mechanism and immune function, which may together lead to a higher risk of POI and other complications (11). Body mass index has also been previously reported to relate to the development of POI after cardiac surgery, including pneumonia, wound infection and mediastinal infection, which strengthens the significance of a scientific diet and proper exercise to maintain a fine body and consequently good health (9, 10, 22, 25). Another significant risk factors for POI identified in this study was chronic renal insufficiency, which was consistent with previous reports (9, 10). Louis and colleagues conducted a prospective multi-institutional cohort study to explore risk factors for mediastinal infection after cardiac operations, finding that higher body mass index and creatinine was significantly associated with the development of mediastinal infection by multivariable analysis (10). A recent review conducted in patients undergoing cardiovascular surgery concluded that advanced age, chronic kidney disease and body mass index were significantly associated with the occurrence of pneumonia (11). In addition, depression was identified as one of the independent risk factors for POI by multivariable analysis in this study, indicating that mental state can also significantly affect the prognosis of the disease. Some other risk factors for POI after cardiac surgery have also been previously reported in the literature but was not identified as independent predictors in our analysis, such as smoking and CPB time (11). This may be caused by the difference in study populations and thus partially reflected the significance and necessity of this study.

The relationship between various infections and short-term outcomes after cardiac surgery has been widely reported in the literature and well recognized in clinical practice (7–9). Joseph and colleagues reported that POI was independently associated with higher risks of mortality, non-home discharge, 90-day readmission and cost increase, and they found that the greatest incremental impact on patient-level and annual cohort costs was due to pulmonary infections (7). Nicolas and colleagues conducted a retrospective analysis of prospectively collected data to study the mortality fraction due to POI in patients undergoing cardiac surgery, finding that patients with POI had significantly higher in-hospital mortality than patients without POI, and pneumonia, bloodstream infection and Pseudomonas aeruginosa infection were each independently associated with increased in-hospital mortality (8). Tamayo and colleagues conducted a prospective observational study in patients undergoing cardiac surgery to identify the impact of POI on patient mortality, finding that the ICU stay was significantly longer and the 90-day survival rate was significantly lower in patients with POI and POI constituted the main independent risk factor for death after the first postoperative week, with 6.23-fold increased risk (9).

Studies focused on a single type of infection have yielded similar results (10, 13, 26, 27). Likosky and colleagues conducted a large-scale multicenter study to examine the relationship between pneumonia and 90-day episode payments and outcomes in patients undergoing CABG and valve surgery, finding that pneumonia was significantly associated with higher 90-day episode payments, longer postoperative length of stay, more frequent discharge to postacute care, and higher rates of 30-day mortality and 90-day readmission (26). Howitt and colleagues conducted a prospective study to assess the incidence and outcomes of sepsis in patients in a cardiac ICU after cardiac surgery, finding that sepsis with both suspected infection and proven infection were associated with increased length of ICU stay and higher 30-day mortality risk, and patients meeting the criteria for septic shock had significantly longer ICU stay, higher 30-day mortality and lower 2-year survival rate than those who suffered sepsis without septic shock (13). Kobayashi and colleagues conducted a multicenter retrospective case-controlled study to examine the socioeconomic effects of surgical site infection after cardiovascular surgery, finding that patients with surgical site infection had significantly longer postoperative hospital stay and higher health care expenditure (13). Louis and colleagues also reported that mediastinal infection after cardiac operations was associated with substantial increases in length of stay, readmission and mortality (10).

Although the relationship between POI and short-term outcomes have been widely examined and reported, studies on the relationship between POI and long-term outcomes after cardiac surgery remains limited. In this study, we found that patients had significantly higher risks of all-cause death, cardiovascular death, HF death, all-cause hospitalization and composite endpoints in patients with POI during follow-up, which was consistent with the limited current literature (24). Roberto and colleagues recently conducted a study-level meta-analysis including 407,829 patients to evaluate the impact of deep sternal wound infection on short- and long-term clinical outcomes, finding that deep sternal wound infection was associated with longer postoperative hospitalization, higher risks of stroke, myocardial infarction, respiratory failure, renal failure, overall mortality, in-hospital mortality, and follow-up mortality during a mean follow-up of 3.5 years.

Another important finding in this study was that although the all-cause mortality was significantly increased in patients with POI during the whole follow-up, the impact of POI on the increased risk of death was mainly reflected in the first postoperative year and the impact was no longer significant during follow-up after the first year. This was similar to the results reported in previous studies (13). In the findings of Howitt and colleagues, although the overall 2-year survival rate was lower in patients with sepsis, the greatest difference in mortality was observed in the first 12 months after surgery and the difference between patients with and without sepsis was no longer statistically significant when only patients surviving more than a year were included in the analysis (13). This relatively short-term effect on risk was consistent with what has been observed in clinical practice, which may be due to the fact that the organ dysfunction that led to severe POI often progressed to organ failure in those had already been physiologically stressed by their operations and patients may recover from these critical complications or die within a relatively short period.

Although there have been many previous studies on POI after cardiac surgery, the current study was still unique and the findings may have potential implications for clinical practice and future research. To the best our knowledge, this was the first multicenter large-scale study that focused on POI after ischemic cardiomyopathy surgery worldwide, which identified the risk factors of POI and clarified its influence on short-term and long-term clinical outcomes. These findings may be helpful for individualized risk estimations, perioperative management and clinical decision-making, which may have certain guiding significance for clinical practice.

## Limitations

Several limitations existed in the present study. First, as a *post hoc* analysis of the STICH/STICHES trial, only patients assigned to surgical treatment and undergoing surgical operations actually were included and analyzed in this study. Patients assigned to medical treatment but undergoing CABG eventually were not included, which may cause some information loss and influence the final analysis results. Second, some factors that may significantly influence the development of POI were not collected and analyzed, such as blood transfusion and medication usage. Third, the 10-year follow-up was completed only in patients assigned to hypothesis 1, and the follow-up was not extended and ended at year 5 in patients assigned to hypothesis 2, which may produce a great deal of censored values and thus influence the analysis results. Fourth, the primary endpoint of this study was POI, which included all types of major infections after surgery; however, further classification of these infections and separate analysis of each type was not performed. Fifth, there has been some progress in medical technology and life support equipment these years, therefore, the impact of POI on prognosis may be different from what it is now due to the fact that the STICH/STICHES trial was conducted more than a decade ago. Sixth, the STICH/STICHES trial was conducted in multiple countries and centers and some centers may only perform very few surgeries, which may lead to significant heterogeneity among different centers, thereby affecting the generalizability of the study findings. Seventh, due to the *post hoc* nature of the analysis, this study cannot avoid the inherent limitations of this type of research and the influence of unknown potential confounding factors.

## Conclusions

POI was prevalent after surgery for ischemic cardiomyopathy, closely associated with higher risks of poor short-term and long-term outcomes. The effect of POI on all-cause death was mainly reflected in the first postoperative year, and was no longer significant during follow-up one year later. Six significant predictors for POI was identified, including age, body mass index, depression, chronic renal insufficiency, Duke CAD Index, and mitral valve procedure. To our knowledge, this was the first report that clarified the relationship between POI and long-term prognosis and the predictors for POI after surgery for ischemic cardiomyopathy worldwide, which may have certain guiding significance for clinical practice.

## Data Availability

Publicly available datasets were analyzed in this study. This data can be found here: https://biolincc.nhlbi.nih.gov/.
